# The Efficacy of Tai Chi and Stretching Exercises Based on a Smartphone Application for Patients With Parkinson's Disease: A Protocol for a Randomized Controlled Trial

**DOI:** 10.3389/fneur.2021.731606

**Published:** 2021-10-28

**Authors:** Renyan Ma, Yuning Hou, Yiyin Zhang, Muyang He, Song Gao, Keneilwe Kenny Kaudimba, Kaiqing Lin, Lingjing Jin, Tiemin Liu, Ru Wang

**Affiliations:** ^1^School of Kinesiology, Shanghai University of Sport, Shanghai, China; ^2^Shanghai Medical College, Fudan University, Shanghai, China; ^3^Department of Neurology, Tongji Hospital, Tongji University, Shanghai, China; ^4^State Key Laboratory of Genetic Engineering, School of Life Sciences, Fudan University, Shanghai, China

**Keywords:** telehealth, smartphone application, Tai Chi, stretching exercises, Parkinson's disease

## Abstract

**Introduction:** Parkinson's disease (PD) is a common neurodegenerative disease that seriously impairs patients' quality of life, and increases the burden of patients and caregivers. Both drugs and exercise can alleviate its motor and non-motor symptoms, improving the quality of life for PD patients. Telehealth, an increasingly popular tool, makes rehabilitation accessible at home, overcoming the inconvenience of traffic and scheduling. Care-PD is a phone application designed for rehabilitation training, which provides Tai Chi and stretching exercises through tutorial videos as well as an online evaluation system. In this protocol, we will explore the efficacy of Tai Chi and stretching exercises as a PD rehabilitation therapy based on the smartphone application Care-PD.

**Methods and Analysis:** A double-blind, parallel randomized controlled trial will be conducted in this study. The recruitment, intervention, and evaluation processes will be implemented through the Care-PD application. Persons with PD will fill out questionnaires on Activities of Daily Living (ADL), upload the latest case report, and sign the informed consent form in the application. Afterward, doctors and researchers will screen and enroll 180 participants who will be randomly (1:1:1) assigned to Tai Chi group, stretching exercises group, or control group. The subjects will participate in a 1-h exercise session three times per week for 12 weeks, ending with another 4 weeks of follow-up study. Each exercise session includes 10 min of warm-up, 45 min of exercise, and 5 min of cool-down. The primary outcomes are Motor Aspects of Experiences of Daily Living and the 39-item Parkinson's disease Questionnaire. The secondary outcomes include the 9-item Wearing-Off Questionnaire, the Freezing of Gait Questionnaire, the Caregiver Strain Index, Non-motor Experiences of Daily Living, ADL, and Morse Fall Scale. All assessments will be performed at baseline, week 12 and 16.

**Discussion:** Care-PD integrates subject recruitment, intervention, and evaluation, providing a new perspective on clinical rehabilitation for persons with PD. This study will evaluate the efficacy of Tai Chi and stretching exercises on patients' quality of life and disease progression based on a smartphone application. We aim to provide a new rehabilitation training platform for persons with PD.

**Ethics and Dissemination:** This study was approved by the Scientific Research Ethics Committee (102772020RT132) of Shanghai University of Sport. Data collection begins after the approval of the ethics committee. The participants must sign an informed consent form before enrollment. The results will be published in relevant journals, seminars, and be disseminated among rehabilitation practitioners and patients with PD.

**Clinical Trial Registration:** Chinese Clinical Trial Registry, identifier [ChiCTR2100042096]. Registered on January 13, 2021.

## Introduction

Parkinson's disease (PD) is a complex and evolving neurodegenerative disease (1–3). The causes of PD have not yet been fully elucidated. The latest research indicates that gene mutation, environmental exposure, and the presence of Lewy bodies may cause PD (4, 5). The incidence of PD is low before the age of 50 but increases rapidly with age, peaking at approximately the age of 80. In most studies, the incidence ratio of males to females is about 1.3–2.0 (6).

Currently, PD affects the daily lives of nearly 6.1 million people worldwide (4). In China, the incidence of PD is rising sharply as population aging aggravates (7). It is estimated that the number of PD patients will increase to 4.94 million by 2030, accounting for nearly half of the cases worldwide. This may impose a considerable burden on the nation's economy and medical system (8). The symptoms and signs of PD can be categorized as motor and non-motor symptoms. Motor symptoms include tremor, stiffness, slow movement, postural instability (1), and freezing of gait (FOG). Non-motor symptoms include cognitive impairment, pain, fatigue, mental symptoms (depression, anxiety), and sleep disorders (9, 10). PD not only severely affects patients' quality of life (QoL) but also increases caregivers' psychological pressure and physical burden (11).

The standard treatments for PD are pharmacologic (levodopa) or non-pharmacologic (exercise, speech therapies) (12). Pharmacologic therapy helps alleviate motor symptoms. However, long-term pharmacologic therapy is accompanied by motor complications, such as dyskinesia and wearing-off phenomena (13). In severe cases, the progression of PD can impair patients' cognitive function (14). Therefore, finding a safer approach is of urgent necessity.

Studies have shown that exercise not only improves cardiopulmonary function, immune function (15), and psychological states but also prevents depression, PD, and Alzheimer's disease (16–18). However, most types of exercise are difficult to popularize among the elderly, especially those with PD.

Tai Chi, a popular exercise among the elderly, is slow-paced and fluent (19). In recent years, the positive effect of Tai Chi on PD symptoms has aroused widespread interest. Hackney et al. presented that Tai Chi improved the balance and mobility of patients with PD (20). Li et al. (21) also found that Tai Chi effectively reduced balance disorders, improved functional capacity, and reduced the incidence of falls in patients with PD. Recently, research indicates that Tai Chi also has a positive effect on cognitive function, sleep quality, and mental health (21–24). However, Shinichi Amano et al. presented that class-based Tai Chi exerted no significant effect on gait or disability rates. According to a systemic review and meta-analysis of seven randomized controlled trials, Tai Chi effectively reduced the incidence of falls (22). In contrast, another meta-analysis suggested no conclusive evidence supporting that Tai Chi can significantly improve the gait velocity and gait endurance (25, 26). Thus, the efficacy of Tai Chi on PD remains to be determined and may be related to the compliance of patients (27).

Tai Chi is typically practiced in groups under the guidance of a coach with rich experience. Unfortunately, the COVID-19 epidemic prevented persons with PD from participating in outdoor activities, possibly worsening their motor and non-motor symptoms (28). Therefore, it is urgent to devise an innovative rehabilitation method accessible to patients at home.

Telehealth based on smartphone applications (app) has developed rapidly, becoming increasingly popular during the COVID-19 pandemic (29). Smartphone apps do not require face-to-face communication between doctors and patients, thereby maintaining social distancing and preventing the spread of the virus (30). Telehealth makes rehabilitation accessible at home, overcoming the inconvenience of traffic and scheduling, and also alleviates the financial burden for patients (31, 32). Telehealth provides a new perspective on home rehabilitation for patients with PD.

Until now, there have been few studies dedicated to home rehabilitation training based on smartphone apps for patients with PD. The Merrill R Landers team created the 9zest app, providing various types of exercise videos, ranging from aerobic exercise, resistance exercise, to yoga and balance training. Their study confirmed the feasibility and safety of app-based exercise (33). However, it lacked an evaluation program that tracks the efficacy of patients' exercise in real time. To overcome the limitations mentioned above, our team developed the Care-PD app, providing home rehabilitation training for patients with PD. Care-PD incorporates Tai Chi, stretching exercises videos, as well as the Self-Assessment module. However, randomized controlled trials on Tai Chi and stretching exercises based on the app are yet to be conducted. This study will conduct recruitment of persons with PD, exercise intervention, and outcome evaluation solely based on the Care-PD app (34).

This study aims to determine the practicability of recruiting subjects, conducting exercise intervention, and evaluating outcomes using a smartphone app only. Additionally, we will explore the effect of Tai Chi and stretching exercises on PD progression. This is the first online randomized controlled trial entirely based on a smartphone app in China. We hypothesize that compared to stretching exercises, Tai Chi is more effective in improving QoL and disease progression.

## Methods and Analysis

### Trial Design

**Figure 1:**
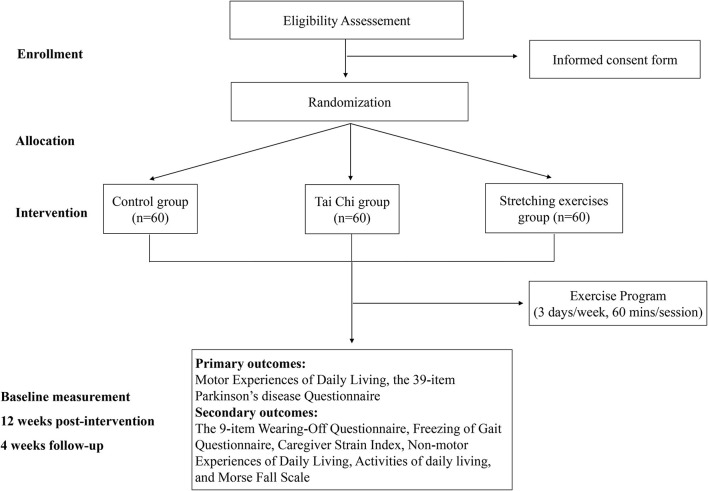
Workflow of the study. Eligible subjects will be randomly divided into Tai Chi group, stretching exercises group, and control group, and evaluated three times.

This study is a double-blind, parallel controlled trial based on a smartphone app. “SPIRIT 2013 Statement Defining Standard Protocol Items for Clinical Trials” is followed in this randomized controlled trial. Each subject will participate in a 12-week Tai Chi and stretching exercises program (Figure 1). Research data will be collected three times. First, sociodemographic information (e.g., age, sex, educational level, and diagnosis certificate or recent medical records from hospitals), and baseline measurement results will be collected. The second data collection will be at 12 weeks after baseline. The third data collection will be at 16 weeks after baseline. The research will start in October 2021 and end in March 2022.

### Participants

We will conduct online recruitment (e.g., WeChat) and post advertisements in communities and hospitals to recruit more participants. The researchers in charge of recruitment will remotely guide participants through the registration process. The subjects with PD interested in our research will sign up for the phone app.

Before enrollment, participants will be comprehensively evaluated based on the following items: (1) diagnosis certificate or recent medical records uploaded in Care-PD (Supplementary Figure 1A), (2) Activities of Daily Living (ADL) index scoring (35, 36), and (3) remote video conference evaluation conducted by professional neurologists. The video conference utilizes two cameras (the primary camera and the secondary camera) to remotely monitor the subjects' performance.

We plan to recruit 180 participants who will be divided into three groups using a 1:1:1 randomization formula: Tai Chi group, stretching exercises group, and control group. Every eligible subject will sign the informed consent form in the app (Supplementary Figure 1B).

The inclusion criteria for subjects with PD are:

(1) Diagnosis of idiopathic PD,
(2) Age of 40 to 75 years with the ratio of males to females ~1–1,
(3) Currently receiving pharmacologic treatment (levodopa) under stable conditions,
(4) Scoring over 55 in ADL index,
(5) Willingness to cooperate with treatment and sign the informed consent form.

The exclusion criteria are:

(1) Diagnosis of typical PD,
(2) Inability to stand for 45 min or complete exercise training,
(3) High risk of falls in the next 6 months indicated by Morse Fall Scale (MFS) (37),
(4) Severe cognitive or mental disorders verified through remote video conference with the help of caregivers,
(5) Inability to use a smartphone or lack of assistance from family members or caregivers to do so.

### Recruitment

The Care-PD app includes eight modules (37). In this study, we mainly use the modules of Research and Recruitment, Self-Assessment, Adverse Report, Exploratory Research, and Medication Recording. The Research and Recruitment module is designed to recruit persons with PD online, while the Self-Assessment module simulates the evaluation process. The Adverse Report module is used for recording adverse events. The Exploratory Research module simulates exercise intervention, providing both Tai Chi and stretching exercises videos. The Medication Recording module is used for recording patients' daily medicine use.

**Figure 2:**
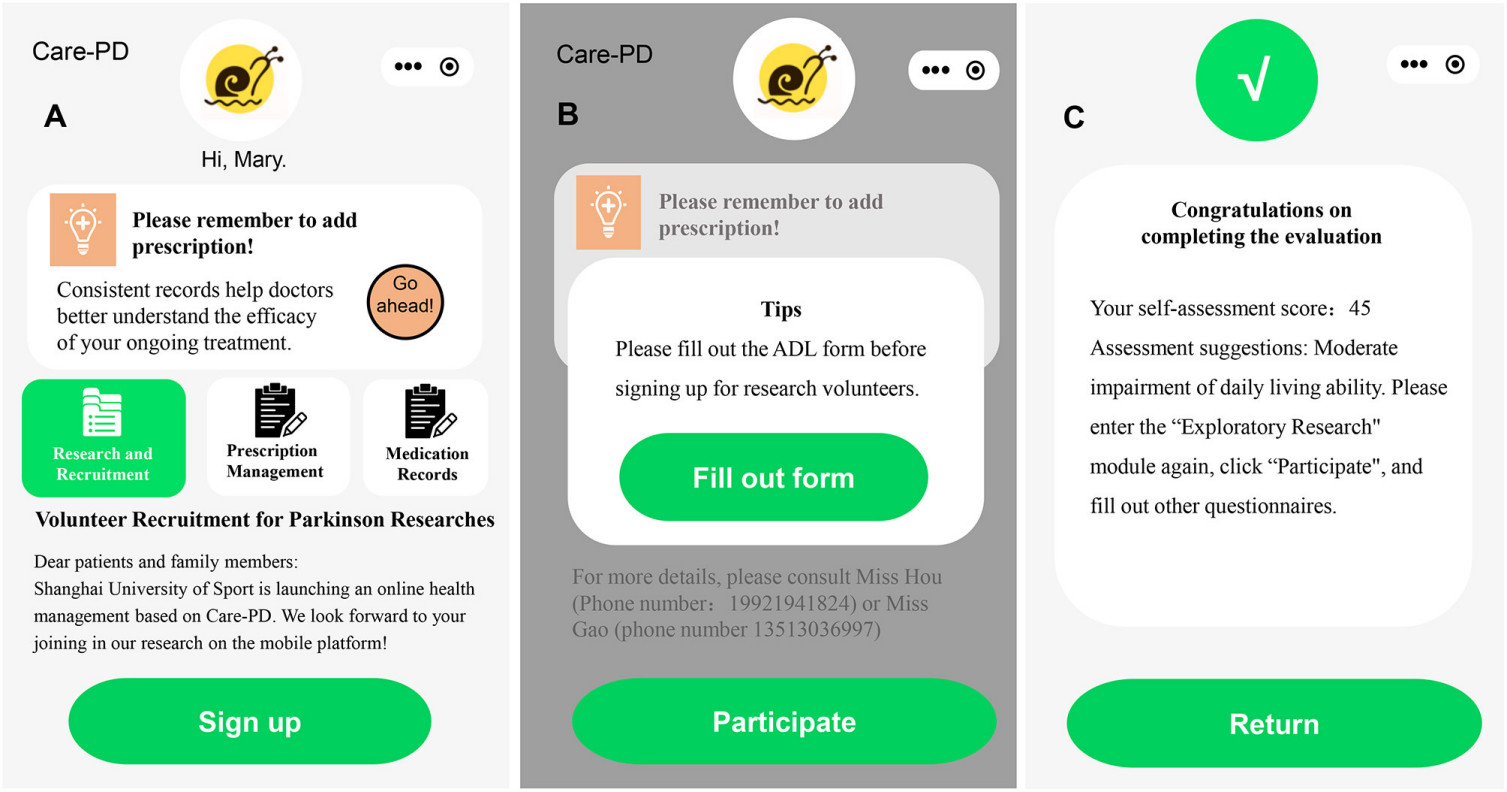
Recruitment information in the app. **(A)** Advertisement of Volunteer Recruitment for Parkinson's Researches, **(B)** the interface of filling out the ADL form, and **(C)** the interface of completing the ADL form.

The Care-PD app will be supervised by neurologists from Shanghai Tongji Hospital and researchers from the Shanghai University of Sport. The patients will complete the form on sociodemographic information, ADL, and sign the informed consent form. At the same time, the administrators, including doctors and researchers, will evaluate and decide whether the subjects fit the criteria. After evaluation, eligible subjects will enter the Research and Recruitment module and be randomly divided into three groups: Tai Chi group, stretching exercises group, and control group (Figure 2). The administrators play an important role in the intervention. First, they evaluate and screen the basic information of subjects. Second, they assist subjects or their caregivers with the installation and registration of the Care-PD app. Last, they supervise the participants' exercise progress and serve as training instructors. All assessments are completed by patients with or without the assistance of the caregivers. Participants can contact the administrators at any time during the intervention period.

### Participant Timeline

As shown in Table 1, the research will be conducted according to the following timeline. First, the subjects will fill out the ADL form and the informed consent form. After evaluation and screening by administrators, persons with PD who fit the inclusion criteria will continue to complete baseline evaluation consisting of eight questionnaires. Then, they will be divided into three groups and participate in a 12-week exercise program. At the end of the program, subjects will complete the evaluation questionnaires. For the next 4 weeks, a follow-up study without exercise intervention will continue. Adverse events will be recorded from week 0 to 16. The questionnaires, same as the ones at baseline, will be filled out again at the end of week 16.

**Table 1:** The time points of data collection.

		**Baseline T0**	**Intervention**	**Intervention end**	**Follow-up**	**Follow-up**
**Assessment**	**Recruitment**	**(Week 0)**	**period**	**T1**	**period**	**end T2**
			**(Week 0–12)**	**(Week 12)**	**(Week 12–16)**	**(Week 16)**
M-EDL		√		√		√
PDQ-39		√		√		√
WOQ-9		√		√		√
FOGQ		√		√		√
CSI		√		√		√
nM-EDL		√		√		√
ADL	√	√		√		√
MFS		√		√		√
Adverse events			√		√	

*“√” means the outcomes will be evaluated in this period. M-EDL, Motor Experiences of Daily Living; PDQ-39, 39-item Parkinson's disease Questionnaire; WOQ-9, The 9-item Wearing-Off Questionnaire; FOGQ, Freezing of Gait Questionnaire; CSI, Caregiver Strain Index; nM-EDL, Non-motor Experiences of Daily Living; ADL, Activities of Daily Living; MFS, Morse Fall Scale*.

### Allocation and Randomization

Randomization is performed with online software (http://www.randomization.com). Participants will receive a number corresponding to Tai Chi group, stretching exercises group, or control group in a randomized controlled table. The allocation result is hidden using a sealed envelope (sequential number). No researcher involved in this study will perform randomization. Researchers responsible for randomization are not involved in the recruitment of subjects, exercise intervention, or outcome evaluation. Similarly, subjects are instructed to withhold the allocation result.

### Intervention

All subjects will take medicine according to prescriptions and record the use of medicine in the Medication Recording module (37). Exercise videos of Tai Chi and stretching exercises will be uploaded to the Exploratory Research module in advance. Each Tai Chi and stretching exercises session consists of 10 min of warm-up, 45 min of exercise, and 5 min of cool-down. The program will last for 12 weeks, with each week containing three sessions of 1-h long exercises, followed by 4 weeks of follow-up without intervention. To ensure safety, subjects will be asked to clear up the surroundings in advance with the assistance of caregivers.

**Figure 3:**
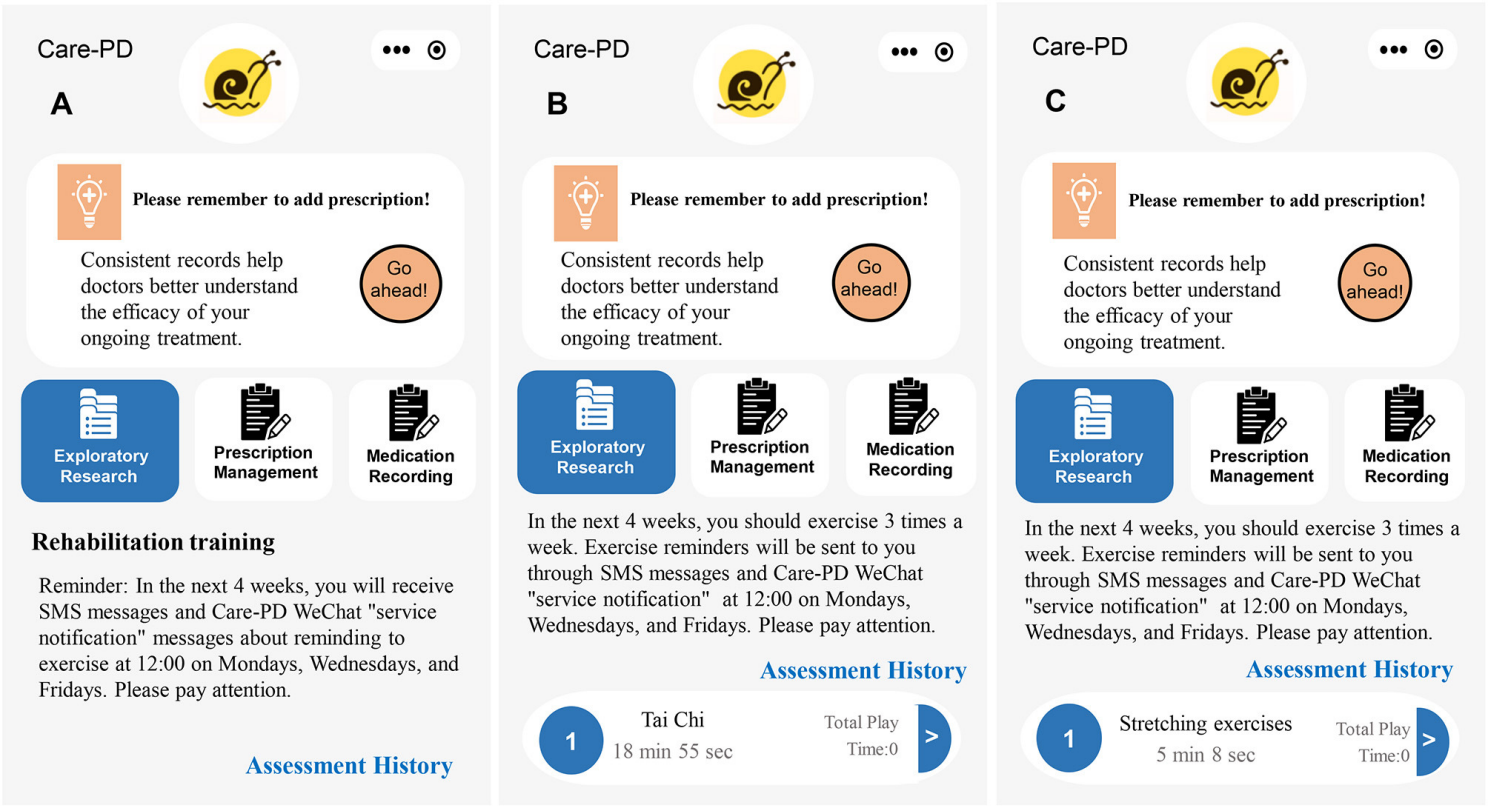
The intervention is based on the Care-PD. **(A)** Control group, **(B)** Tai Chi group, and **(C)** stretching exercises group.

In addition, WeChat and Tencent Meeting will be used for communicating with the participants. Before each intervention, Care-PD will send a notification as well as a text message to remind the participant to exercise according to schedule. During the intervention, a video conference will be initiated that the subjects then join with the camera on. The entire process of the exercise intervention will be supervised and recorded. The researchers will inform subjects on the techniques of exercise, safety precautions, and emergency measures in case of adverse events. At the end of the intervention, the researchers will remind the subjects to log in and inquire about changes in their physical and psychological conditions. The specific plan of intervention is shown in Figure 3. The detailed moves of Tai Chi and stretching exercises are presented in Table 2.

**Table 2:** The program of Tai Chi and stretching exercises.

**Type of exercise**	**Descriptions of program**
Tai Chi	1. Preparing and starting form
	2. Up and down form
	3. Bend over form
	4. Curled spine form
	5. Cross wrists form
	6. Go through palms form
	7. Standing on one leg
	8. Flying form
	9. Opening hands and closing hands
	10. Closing form
Stretching exercises	1. Stretching exercises
	2. Neck and upper limb exercises
	3. Moves of waist, abdomen, and lower limbs
	4. Hand moves
	5. Mouth and facial moves
	6. Exercise on the mat

### Tai Chi Group

Tai Chi group joins a 12-week Tai Chi training program that utilizes the Medication Recording module in Care-PD to instruct the use of medicine. Simple Tai Chi, designed by martial art specialists based on the characteristics of PD symptoms, includes 10 exercises: (1) the preparatory and rising, (2) lifting, (3) leaning, (4) spine, (5) wrist-wrapping, (6) palming, (7) freestanding, (8) flying, (9) opening and closing, and (10) retracting positions. The total duration of Tai Chi is 18 min and 55 s. The detailed moves of Tai Chi have been introduced in a previous article (34). The program is remotely supervised by an experienced Tai Chi specialist without the involvement of researchers. The aim of the first 2 weeks of intervention is for the subjects to master every move of the exercise. From week 3 to 12, subjects will perform the full Tai Chi exercise 2–3 times, with 5–10 min of rest in between depending on the fluency of exercises. The number of repetitions is determined by the physical fitness of subjects.

### Stretching Exercises Group

The stretching exercises group joins a 12-week stretching exercises program that also uses the Medication Recording module to instruct the use of medicine. The stretching exercises are designed by a team of experts from Shanghai Tongji Hospital based on the characteristics of PD. It consists of six parts: (1) stretching, (2) neck and upper extremity exercise, (3) waist, abdomen, and lower extremity exercise, (4) hand exercise, (5) mouth and face exercise, and (6) mat exercise. The total duration of the stretching exercises is 14 min and 18 s. Detailed moves of stretching exercises are shown in Supplementary Table 1.

### Control Group

The modules of the Care-PD app available to the control group are consistent with those of Tai Chi group and stretching exercises group except for training programs. The control group will have access to the Medication Recording module but not the exercise videos. After the trial, Tai Chi and stretching exercises videos will be available to all participants in the control group.

### Outcome Assessment

**Figure 4:**
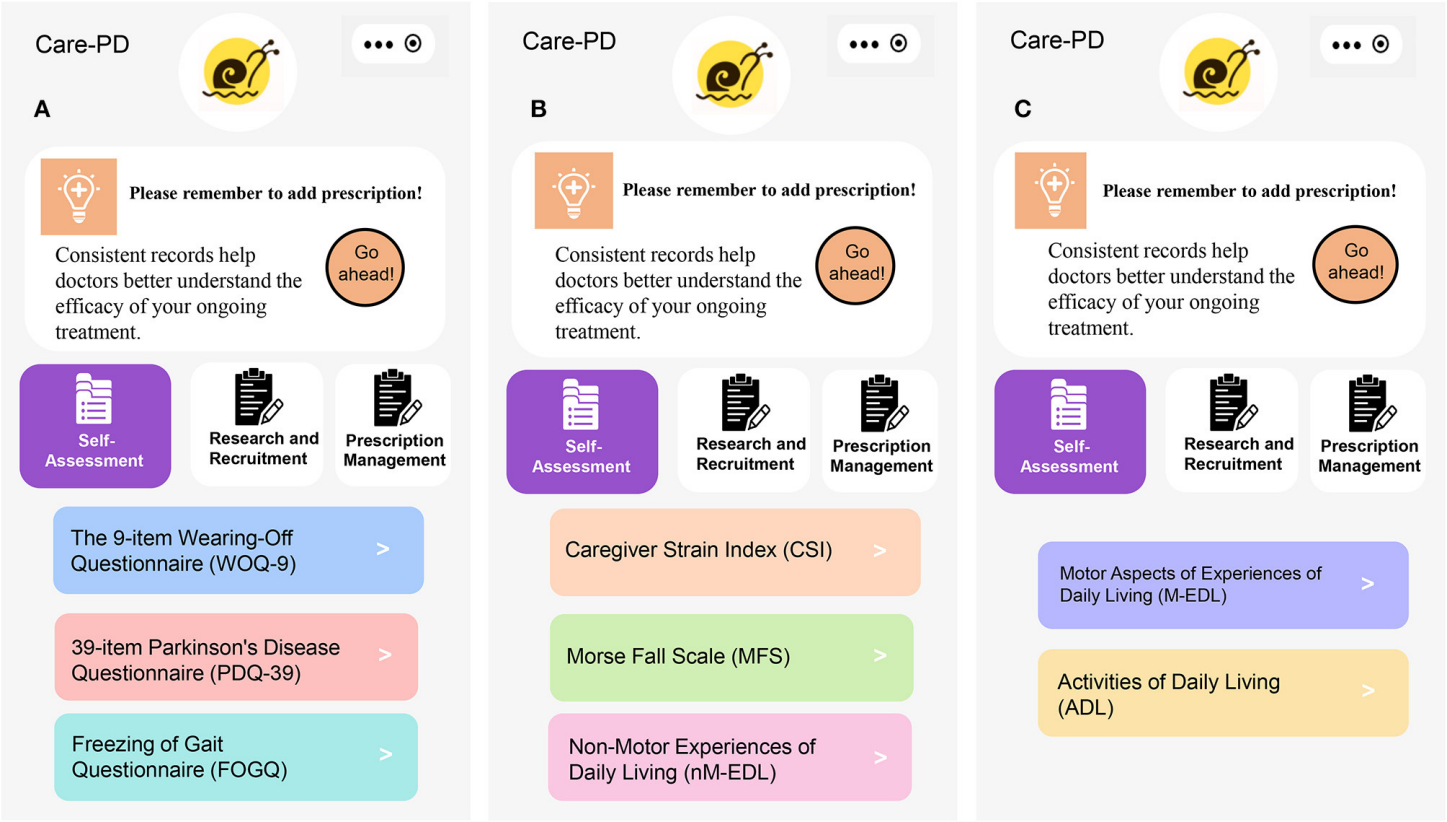
Questionnaires of outcome assessment. **(A)** WOQ-9, PDQ-39, and FOGQ, **(B)** CSI, MFS and nM-EDL, and **(C)** M-EDL and ADL.

The Exploratory Research module includes eight evaluation questionnaires (Figure 4). The outcomes will be assessed at baseline, the end of exercise intervention, and the follow-up period. Patients or their caregivers will be asked to fill out the questionnaires accurately.

### Sociodemographic Information

Sociodemographic information including gender, age, height, marital status, living status, education level, and working status will be collected at baseline.

### Clinical and PD-Related Information

Medical history, the number of falls in the past 6 months, lengths of the illness, disease progression, and medication use will also be collected.

### Primary Outcome Measures

#### Motor Aspects of Experiences of Daily Living

The M-EDL is the second part of the Movement Disorder Society–sponsored revision of the Unified Parkinson's Disease Rating Scale (MDS-UPDRS), explicitly designed for measuring cognitive impairment, thought disorder, depression, and motivation (38). It contains 13 items, including speech, saliva and drooling, chewing and swallowing, handwriting, doing hobbies and other activities, eating tasks, tremors, dressing, hygiene, turning in bed, getting out of bed, walking and balance, and freezing. The total score of the M-EDL is 52, and the score of each item ranges from 0 (normal) to 4 (severe) (39, 40). The high score of the M-EDL indicates bad physical fitness. All items have five responses with uniform anchors of 0 = *normal*, 1 = *slight*, 2 = *mild*, 3 = *moderate*, and 4 = *severe*. Total score ranges from 0 to 52, with higher scores reflecting severer conditions.

#### The 39-Item Parkinson's Disease Questionnaire

The PDQ-39 is a 39-item self-report questionnaire, which assesses how often PD patients experience difficulties across eight quality of life dimensions including mobility, activities of daily living, emotional well-being, and stigma, social support, cognition, communication, and bodily discomfort (41). The questionnaire uses a 5-point ordinal scoring system (42) with each dimension total score ranging from 0 to 100, where lower scores represent better QoL (43).

### Secondary Outcome Measures

#### Non-motor Experiences of Daily Living

The nM-EDL is the first part of the MDS-UPDRS (38, 44). The nM-EDL contains eight questions, covering sleep status, tiredness, pain, and other sensations, dysuria (frequent urination, urgent urination or incontinence), constipation, dizziness, and fatigue while standing. The scoring method is the same as the M-EDL. The higher the score, the severer the symptoms.

#### The 9-Item Wearing-Off Questionnaire

The WOQ-9 is capable of diagnosing 95% of wearing-off (WO) after dopaminergic therapy (45). It consists of five questions on motor symptoms and four questions on non-motor symptoms (46). A sum score of the WOQ-9 higher than 2 indicates the occurrence of WO (47).

#### Freezing of Gait Questionnaire

FOG causes falls, limb fractures, and is frequently observed in long-term and terminal PD (48). The FOGQ is used for evaluating the freezing of gait which occurs when patients temporarily feel glued to the ground before stepping forward, accompanied by tremor or drag. Higher FOGQ scores indicate worse conditions of FOG.

#### Caregiver Strain Index

The CSI is a 13-item questionnaire designed to assess the level of strain experienced by caregivers (49), and indirectly reflects the physical fitness of subjects. Answers are either affirmative (1 point) or negative (0 point). The total score of the CSI ranges from 0 (no pressure) to 13 (maximum pressure) (50). A higher score reflects a higher level of caregiver strain. A total score no lower than 7 indicates that the caregiver is under high pressure.

#### ADL

The ADL form is an essential method for evaluating the level of disability and independence of persons with PD (51, 52). The ADL form contains 10 items, including behavior, cognition, and motor skills. The total score ranges from 0 to 100, with higher scores indicating worse ADL.

#### MFS

The MFS is designed to assess the risk of falling in the next 6 months. The main questions include “Did you have a fall last year? Has FOGQ happened in the past month? Does it take you more than 3.6 s to walk 4 m in a relaxed manner?” The total score ranges from 0 to 125, with higher scores indicating higher risks of falls.

### Adherence Promotion

Participant adherence is measured by the rate of recruitment and retention, online time, and other adherence performance. Coaches and doctors are responsible for supervising and managing the participants:

(1) Before each exercise, the attendance rate will be counted.
(2) During the exercise, log-in time in the video conference and Care-PD app will be recorded, respectively. Moreover, coaches will supervise the exercise through video conference which will be recorded and reviewed subsequently.
(3) Before and after the intervention, the time spent on filling online questionnaires will also be recorded.

In addition, the subjects will receive verbal encouragement and humanistic care. We plan to reward participants for following instructions to improve their adherence to the program. Immediate results from the assessment module in the application will also create a positive feedback loop that stimulates participant adherence.

### Adverse Events

Subjects need to record daily adverse events, including falls, muscle soreness, injuries, and dizziness. Once an adverse event occurs, they should contact the doctors or researchers as soon as possible and call the emergency number if necessary. To ensure that subjects complete the record of adverse events, the doctors and the researchers will verify the record every time they contact the participants.

### Sample Size

The calculation of sample size is based on a previous study finding a power size of 0.8 with 45 participants in each group (21). We use G^*^Power 3.1.9.7 software to calculate the number of subjects (53, 54). A sample of 180 subjects (60 subjects in each group) is required to realize the effect size dose of 0.25 with 0.05 alpha, and the drop-off rate of 30–35% will be considered. The sample calculation utilizes the analysis of variance (ANOVA) analysis of repeated measures among factors.

### Data Collection, Management, and Analysis

All personal information will be kept strictly confidential, such as age and disease progression. The results will be collected and stored in a database protected by passwords.

Data will be obtained from the Care-PD app and presented as mean ± SE. All statistical analyses will be performed in GraphPad Prism and R studio 3.6.3. Staff with rich experience will independently complete the processing and cleaning process of data. Baseline data between groups will use one-way ANOVA for continuous variables and normal distribution, Wilcoxon test for continuous variables and non-normal distribution, and chi-square test for categorical variables. Repeated-measure ANOVA is used to evaluate the efficacy of Tai Chi and stretching exercises on QoL and PD progression over 12 weeks (within-group). A mixed-model repeated-measure ANOVA is used to compare the difference between Tai Chi, stretching exercises, and the control (between-group).

### Ethics and Dissemination

This study was approved by the Research Ethics Committee of Shanghai University of Sport (102772020RT132) and clinical trial (ChiCTR2100042096). All eligible participants will be informed of the research purpose and confidential data processing. We plan to purchase insurance for every subject participating in this study, covering all damages and injuries that may occur.

### Confidentiality

Only permitted researchers have access to data and research design. The data will be strictly confidential during and after the research. We will not share any information with third parties. The relevant members of the Ethics Committee of Shanghai University of Sport will also strictly abide by the confidentiality regulations and avoid any form of data leakage.

## Discussion

Tai Chi is a practicable rehabilitation exercise for PD patients. Previous studies have shown that Tai Chi alleviates balance disorder and other motor symptoms in patients with mild to moderate PD (21). The study of Li et al. (21), which recruits 176 participants for 6 months of Tai Chi and stretching exercises training, demonstrated the positive effect of Tai Chi on balance and motor function for PD patients. A systematic review and meta-analysis showed that Tai Chi effectively improved the incidence of falls (22), balance, motor function, QoL, and depression (55), but not gait velocity, gait endurance for persons with PD (25, 26).

During COVID-19, lockdowns and social distancing restrictions made it difficult to exercise outdoors. However, the rapid development of telehealth has created many new opportunities for home rehabilitation for persons with PD. A recent study has designed a rehabilitation training app for persons with PD, providing various exercise videos, ranging from aerobic exercise, resistance exercise, yoga, to balance training. Although they provided various types of exercise tutorials, the app lacks an online evaluation system with immediate feedback. Patients could not keep track of the effect of their exercises, and it was difficult to adjust the exercise program timely (33). A new protocol was published on the effect of Tai Chi on daily activities and QoL for persons with PD based on the “Shoupa” app. However, the outcome assessments all require the physical interactions between patients and researchers, such as Limits of Stability (maximum excursion and directional control), Berg Balance Scale, Functional Reach Test, Timed Up and Go Test, and 6-Min Walk Test (34).

During the COVID-19 pandemic, face-to-face assessment is risky and inconvenient. Therefore, it is necessary to build an online evaluation platform for home rehabilitation exercises. Care-PD is a home rehabilitation app, providing Tai Chi and stretching exercises videos created by professional coaches and neurologists. Patients can not only exercise under the guidance of tutorial videos but also track disease progression in the app. The most significant difference between our study and the protocol of Gao et al. (34) is that our study is entirely based on the mobile app, which integrates subject recruitment, exercise intervention, and outcome evaluation. Gao et al. introduced the platform of Care-PD in management and rehabilitation for persons with PD. Despite the lack of significant difference between Tai Chi group and the control group, they demonstrated that after 8 weeks of rehabilitation using Care-PD for Tai Chi and medicine management, both the motor and non-motor symptoms of participants improved. Thus, the Care-PD platform is a viable option for home rehabilitation.

This study will explore the effect of Tai Chi and stretching exercises on QoL, motor and non-motor symptoms for persons with PD utilizing the Care-PD app. In the future, we plan to further promote and popularize the Care-PD app among persons with PD. However, there still exists certain limits. First, the majority of PD patients are between 60 and 80 years old and cannot operate mobile phones readily. Second, it is difficult to ensure the consistency of exercise intensity and duration for every participant as exercise intervention is conducted remotely. Last, outcomes can only be assessed through online questionnaires in the app. Physical examinations that require face-to-face contact, such as the 6-Min Walk Test, Timed Up and Go test, Limits of Stability, and Functional Reach Test, cannot be performed.

Moreover, the subjects need to maintain their habitual lifestyles, physical activity, and avoid participating in other exercise programs.

In conclusion, we hope that Care-PD and its exercise and assessment features can reach more PD patients in need of home rehabilitation, ultimately improving their QoL as well as motor and non-motor symptoms. Care-PD will serve as a rehabilitation platform assisting PD patients and caregivers, as well as guides and other materials for the reference of neurologists.

## Ethics Statement

The studies involving human participants were reviewed and approved by Research Ethics Committee of Shanghai University of Sport (102772020RT132). The patients/participants provided their written informed consent to participate in this study.

## Author Contributions

RM and YH prepared and repeatedly revised the manuscript. YZ and SG are responsible for the study design and ethics application. MH, KL, and KK revised the manuscript. LJ, TL, and RW contributed to the development, procedure, and funding for research. All authors reviewed the manuscript and provided final approval for the manuscript.

## Funding

This work was supported by the National Key R&D Program of China (2020YFA0803800, 2018YFC1314700), the National Natural Science Foundation of China (31971097, 31671242), the Construction Project of High-Level Local Universities in Shanghai, China, Shanghai Frontiers Science Research Base of Exercise and Metabolic Health, and Multidisciplinary cross-innovation team of metabolic diseases in Chinese medicine (ZYYCXTD-D-202001).

## Conflict of Interest

The authors declare that the research was conducted in the absence of any commercial or financial relationships that could be construed as a potential conflict of interest.

## Publisher's Note

All claims expressed in this article are solely those of the authors and do not necessarily represent those of their affiliated organizations, or those of the publisher, the editors and the reviewers. Any product that may be evaluated in this article, or claim that may be made by its manufacturer, is not guaranteed or endorsed by the publisher.

## Supplementary Material

Supplementary Figure 1 **(A)** Diagnosis certificate or recent medical records uploaded in the Care-PD, **(B)** Online Informed Consent Form.

Supplementary Table 1 The detailed description of stretching exercises.
