# Supplementary figures and images for: The Efficacy of Tai Chi and Stretching Exercises Based on a Smartphone Application for Patients With Parkinson's Disease: A Protocol for a Randomized Controlled Trial
Source: Front Neurol. 2021 Oct 28;12:731606. doi: 10.3389/fneur.2021.731606 (PMC8581180; doi:10.3389/fneur.2021.731606)

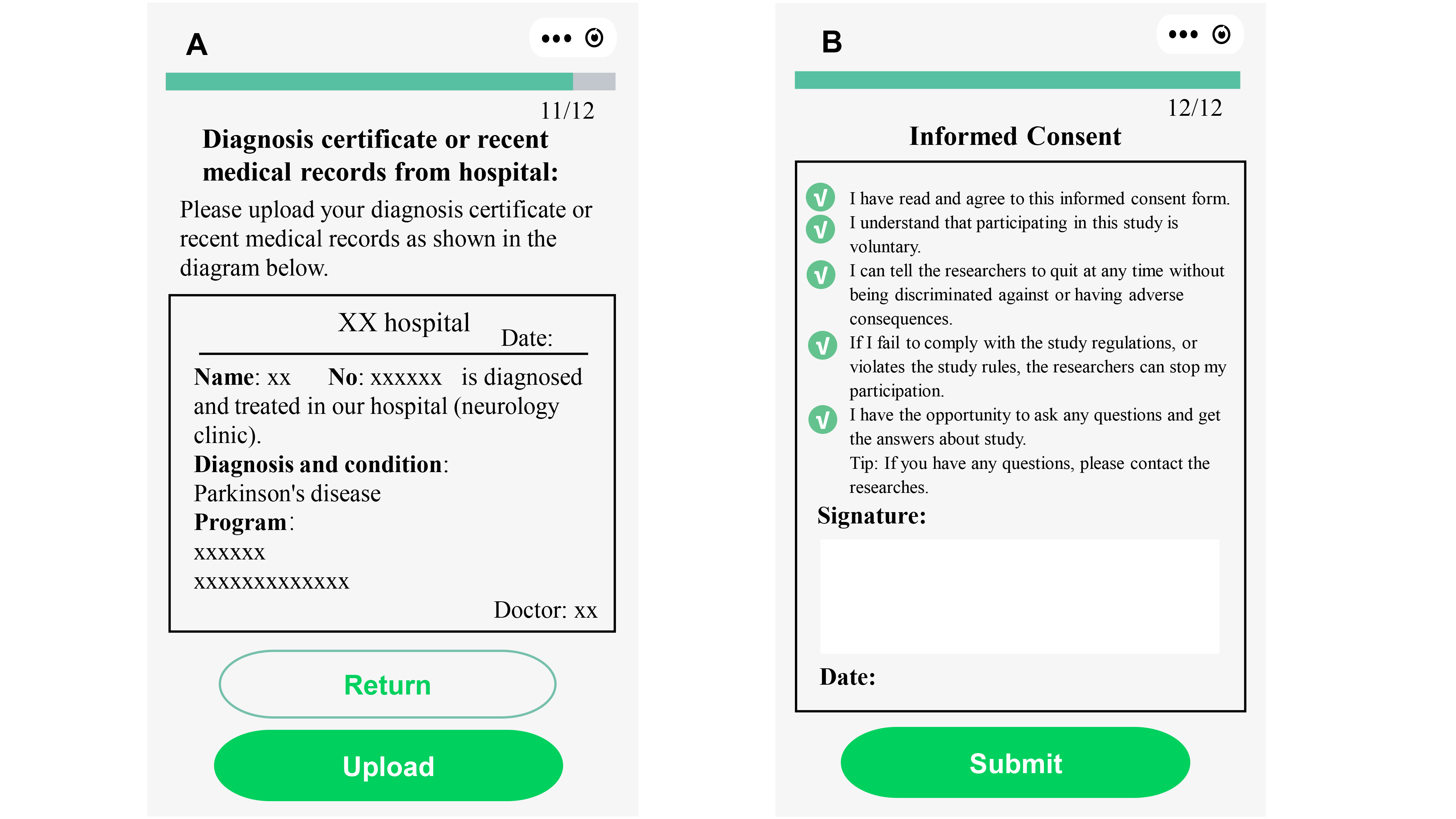

Supplement: Supplementary Figure 1 — (A) Diagnosis certificate or recent medical records uploaded in the Care-PD, (B) Online Informed Consent Form. [file Image_1.TIF]
