# Supplementary material for: The Efficacy of Tai Chi and Stretching Exercises Based on a Smartphone Application for Patients With Parkinson's Disease: A Protocol for a Randomized Controlled Trial
Source: Front Neurol. 2021 Oct 28;12:731606. doi: 10.3389/fneur.2021.731606 (PMC8581180; doi:10.3389/fneur.2021.731606)
Supplement: Supplementary Table 1 — The detailed description of stretching exercises. [file Table_1.DOCX]

Supplementary Material

# Supplementary Table 1. The detailed description of stretching exercises.

| Sections of program | Description |
| --- | --- |
| 1.Stretching | Each move is maintained for 5 seconds, alternately performed 2 times left and right.  a. Keep body upright, look forward, turn head to the front left, then slowly lower head, meanwhile turn head and neck to the other side.  b. Raise arms up, relax after 5 seconds. Straighten arms and stretch them horizontally.  c. Bend the left arm behind the head, try to touch the right shoulder blade, and repeat the action with the right arm.  d. Separate the legs and keep straight, bend down and try to touch the right toe with the left hand, and touch the left toe with the right hand.  e. Take a big step forward with the left leg, set the heel of the right leg on the ground, and press the center of gravity to the left leg until the legs feel stretched. |
| 2.Neck and upper limb exercises | Each move is maintained for 5-10 seconds, 8 repetitions/group.  a. Keep body upright, look straight ahead, retract neck so that chin is close to the skin of the front neck.  b. The body is upright while the upper limbs droop naturally, and the shoulders are shrugged on both sides.  c. With hands on hips, do shoulders circle move, slow down, and try to reach the four directions of front, back, up and down evenly.  d. High-five hands on both sides, and fall from both sides of the body. When falling, keep arms straight, palms down, and try one’s best to lift the back of hands.  e. Spread feet naturally and open arms horizontally, turn around and touch left hand with right hand, then slowly retract right hand along the left arm to chest, then straighten it back to the horizontally opened state, repeating alternately left and right. |
| 3.Move of waist, abdomen and lower limbs | All moves alternate left and right 4 times each for 3-5 seconds/time.  a. Separate legs naturally, hold hands in front of chest, and use the upright position as the axis to make the upper body move around the axis.  b. Set the center of gravity on the right side, and lift left hip up to suspend left foot.  c. Step on the spot and raise thighs as much as possible.  d. On the basis of 3, when lifting the left leg, the right hand touches the left knee.  e. With the position of the left station as the center, use the walking trajectory to draw rice characters, and the strides should be as close as possible.  f. Place a paper box with a suitable height in the front to complete the threshold crossing action. After proficiency, the reverse crossing back action can be completed.  g. Take a small step with the left foot, make the heel stick to the right toe, and then move forward with the right foot to make the right heel stick to the left toe. |
| 4.Hand moves | Alternate the left and right hands on both sides.  a. The wrist rotates around with a stable speed.  b. Simulate the tiger claws in Wu Qin Xi, keep the metacarpophalangeal joints stretched while the interphalangeal joints bend vigorously.  c. Open hands to the number 5, and the palms are about 20 cm opposite each other. Meanwhile, merge hands to the midline, cross fingers and squeeze, quickly loosen and return to the original position once, repeat quickly.  d. Both hands can be completed at the same time or separately, and the thumb and other four fingers can quickly face each other.  e. Push the index finger of the left thumb forcefully, then the right hand completes the same action.  f. Complete numbers 1-9 with both hands simultaneously or individually.  g. The left hand is better than the number 8 and the right hand is better than the number 4. Both hands are converted into the opponent's number at the same time, alternating. |
| 5.Mouth and facial moves | All moves are maintained for 5 seconds, 10 times/group. The strength can be grasped according to the degree of fatigue.  a. Lift both eyebrows vigorously and open eyes.  b. Lift up the corners of the mouth to expose as many teeth as possible.  c. Close lips tightly and puff cheeks hard to make the gas fill mouth as much as possible.  d. When the cheeks are blown, the gas in the mouth can be quickly moved left and right.  e. Cover the teeth with lips, make sure to make a crisp sound after the upper and lower sides are closed tightly.  f. The tip of the tongue presses against the upper jaw and makes a crisp sound.  g. The tip of the tongue moves quickly and alternately between the left and right corners of the mouth.  h. Close the tip of the tongue to the tip of the nose and the lower jaw, alternately. |
| 6.Mat exercise | Alternate left and right, 10 times/group, 5-10 seconds/time, be adjusted appropriately according to personal situation.  a. Lie supine on the bed with legs bent and raise hips.  b. Lie supine on the bed, with legs bent, and after raising hips, straighten left leg.  c. Straighten legs together while lift heels from the bed. Avoid raising legs too high and falling quickly.  d. Lie on the left side, use leg to straighten and lift up sideways.  e. Lying on the side of the bed, with the left leg hanging under the bed, lift the foot to the height of the bed with the force of raising the thigh. |
